# Modeling the integration of ethics, social responsibility, and sustainability in university contexts

**DOI:** 10.1371/journal.pone.0337068

**Published:** 2025-12-09

**Authors:** John William Atehortúa-Mosquera, Iliana Ramírez-Velásquez

**Affiliations:** Faculty of Exact and Applied Sciences, Instituto Tecnológico Metropolitano, Medellín, Colombia; Universitas Islam Negeri Raden Intan Lampung, HUNGARY

## Abstract

The integration of ethics, social responsibility, and sustainability (ERS) into higher education curricula is essential for fostering competencies aligned with the Sustainable Development Goals. This study aimed to develop and validate a structural model to evaluate ERS competencies in higher education. Data were collected from a sample of 418 engineering students at a public university in Colombia, using a Likert-type instrument comprising items distributed across three dimensions. Exploratory factor analysis identified a well-defined three-factor structure, while confirmatory factor analysis confirmed the model’s validity. The structural equation model demonstrated a good fit (CFI = 0.946, TLI = 0.940, RMSEA = 0.047, SRMR = 0.042), and reliability analysis indicated excellent internal consistency (α = 0.914; ω = 0.920 for the higher-order ERS construct). Results showed that ERS significantly explained the first-order dimensions of Ethics (0.620), Sustainability (0.884), and Social Responsibility (0.847). Moreover, ERS predicted academic performance in transversal courses, with standardized coefficients of 0.698 in Science, Technology, and Society (STS) and 0.574 in Environmental Management (p < .001). These findings provide strong empirical support for the hierarchical structure of ERS competencies, confirm their positive association with academic achievement, and highlight the model’s value as a tool to guide curriculum development and policy in higher education.

## Introduction

Ethics, Social Responsibility, and Sustainability (ERS) form a triad that has become a strategic priority in higher education at the global level. Over the past decades, this field has attracted increasing research interest, driven by the urgent need to educate professionals capable of responding to the complex social, economic, and environmental challenges of the 21st century [1]. The interdisciplinary convergence of these three pillars has not only enriched academic discourse but also strengthened the comprehensive development of students, significantly influencing both their academic training and their personal and professional growth [2].

Within this framework, ethics, understood as the study of the principles that guide human conduct toward the common good [3], has gained prominence in higher education, particularly in the preparation of critical and socially responsible citizens. Ethics education should go beyond the mere transmission of norms and rules; it should foster a reflective attitude that enables students to make well-reasoned decisions committed to social and environmental well-being [4]. This ethical approach requires not only conceptual understanding but also the ability to act in real-world contexts, considering the global implications of personal and professional choices.

Social responsibility, in turn, refers to the commitment of individuals and institutions to the collective good, involving a sense of obligation toward the environment, vulnerable communities, and future generations [5,6]. Training in this area seeks to develop not only academic knowledge, but also social and emotional skills that allow students to recognize and address complex issues such as poverty, inequality, or social justice. That’s how universities must assume responsibility for training competent professionals who are conscious citizens, aware of their social and environmental impact [7].

Sustainability refers to development that meets present needs without compromising the ability of future generations to meet theirs. In contemporary higher education, sustainability has become an essential principle [8,9]. Its integration into academic programs goes beyond imparting knowledge about climate change or resource management; it aims to promote attitudes and practices focused on problem-solving, innovation, and collective well-being [10]. This type of training involves not only the development of technical competencies in areas such as circular economy, renewable energy, and environmental management, but also the critical capacity to reflect on each individual’s role in building a more just and equitable future [11].

From this multidimensional perspective, the literature underscores that ethics, social responsibility, and sustainability are foundational axes for designing and implementing innovative educational strategies. These principles not only guide students’ holistic education but also underpin the transversal integration of content in both the formal curriculum and at the mesocurricular level [12]. The mesocurriculum is conceived as an intermediate level within the curricular structure that bridges learning experiences with the formal curriculum, establishing the formative parameters that guide shared learning spaces within higher education institutions. As such, it plays a pivotal role in the comprehensive education of students [13].

Aspects considered at this level include pedagogical guidelines, shared learning objectives across different academic programs, and underlying principles embedded in institutional courses. These are designed to foster cross-disciplinary preparation relevant to various fields [14], particularly in areas such as ethics, sustainability, and social responsibility [15]. The pedagogical value of the mesocurriculum lies in its ability to offer authentic and contextualized learning environments, where students not only apply technical knowledge but also engage with real-world dilemmas that demand critical reflection and value-based action [10].

In this regard, the mesocurriculum not only complements academic learning but also serves as a strategic space for experiencing ERS principles, fostering transformative education that links theory with practice and promotes active and engaged citizenship [16,17]. Thus, the integration of ethics, social responsibility, and sustainability (ERS) should not be understood merely as an isolated pedagogical component but as a comprehensive formative process permeating all dimensions of the educational experience.

This perspective promotes not only the acquisition of technical knowledge but, more importantly, the development of a profound ethical and social consciousness aimed at addressing contemporary challenges with a committed and transformative vision. In this context, student engagement gains significance as it is conceived as the cognitive, emotional, and behavioral involvement of students with their education and their academic and social environment [18], being closely linked to the internalization of ethical values and the construction of critical consciousness [19]. Furthermore, this engagement manifests through active, persistent, and goal-oriented participation [20], fundamental aspects for the appropriation of ERS principles.

Evaluating various concepts aligned with ethics, social responsibility, and sustainability within a context that fosters autonomous learning and student engagement is essential for designing didactic strategies that encourage participation in both academic training and social projection [20,21]. The didactic strategies implemented in course development serve as a fundamental articulating axis in formative processes aimed at the transversal integration of ERS in higher education [2]. Methodologies such as service-learning, project-based learning, communities of practice, and interdisciplinary collaborative experiences have become benchmarks in the literature for their ability to link disciplinary knowledge with the development of ethical values and capacities, social responsibility, and sustainability [16,22].

These strategies promote experiential, reflective, and contextualized learning environments that place ethics, social responsibility, and sustainability at the core of the educational process. In these settings, students assume an active, autonomous, and co-responsible role, not only in constructing knowledge but also in applying it to real scenarios with social and environmental impact [23]. In this sense, the mesocurriculum emerges as an ideal space for incorporating formative dimensions that, although not always structured within study plans, allow for the experience of ethical principles in various contexts, emphasizing the importance of including aspects related to ERS in training processes [15]. Through these experiences, students not only strengthen professional knowledge but also confront real dilemmas that demand responses grounded in ethical values [24]. Thus, the mesocurriculum not only complements technical or disciplinary training but becomes a privileged means to articulate theory with praxis concerning the challenges of ethics, social responsibility, and sustainability [25].

From an integrative perspective, the inclusion of ERS principles in the formal curriculum, adhering to mesocurricular guidelines, highlights the need for training that combines technical knowledge with ethical attitudes and socio-emotional skills. Research concurs that such training should be based on active methodologies, collaborative strategies, and contextualized pedagogical processes capable of involving students in meaningful learning situations [7]. By connecting theory with practice, these strategies enable students to appropriate ERS principles not merely as conceptual content but as action guides that direct their behavior in personal, professional, and civic life.

Reflective learning encourages individuals to contemplate the broader implications of their actions on society and the environment. By adopting an ethical perspective, students prioritize values such as equity, sustainability, and collective well-being in their decision-making processes. In this way, social responsibility becomes fully integrated into learning, transforming it into a means to generate positive societal impact [26,27]. Valid and reliable educational tools and frameworks, such as assessment instruments, play a crucial role in strengthening this process [2,28]. These tools foster and ensure that learning aligns with ethical standards, promoting decisions that contribute to a more just and sustainable world. Through this synergy, learning becomes a transformative experience that drives both individual development and social progress [23,29,30].

Nevertheless, despite the progress achieved, obstacles persist that hinder the full incorporation of ERS into curricula. Among these are the lack of consensus on the most appropriate pedagogical approaches, institutional resistance to curricular transformation processes, and the deficit of instruments that allow for the integrated evaluation of ERS appropriation across different training spaces [28,30]. These limitations can restrict the development of an institutional culture oriented toward ethics, social responsibility, and sustainability, thereby affecting the training of citizens committed to their environment. In response to these barriers, the literature proposes strategies such as curricular reorientation aligned with the Sustainable Development Goals (SDGs), the design of innovative methodologies, and the creation of inclusive and dynamic learning environments [31]. However, all these proposals require instruments that provide information about students’ perceptions regarding knowledge, experiences, and the articulation of their specific training around ERS.

In line with the above, this study contributes to advancing the understanding of the development of ethics, social responsibility, and sustainability (ERS) in higher education through the analysis of the psychometric properties of a self-perception instrument designed to evaluate these dimensions in university students [15]. Within this framework, a model is proposed that relates ERS competencies with parameters established at the mesocurricular level to analyze their integration with the academic performance in common spaces within the institutional training offer. The model’s evaluation was conducted through specific indicators and coefficients [32,33] to determine the incidence and degree of appropriation of ERS competencies among participants.

This work begins with an exploratory factor analysis (EFA) aimed at identifying the underlying structure of the data and exploring the empirical grouping of items into latent factors. Subsequently, a confirmatory factor analysis (CFA) was carried out to verify the adequacy of the proposed model to the observed data. To assess the model’s fit, the chi-square statistic (X²) to degrees of freedom (df) ratio was used, along with other goodness-of-fit indices widely recognized in the literature, such as RMSEA (Root Mean Square Error of Approximation), TLI (Tucker-Lewis Index), CFI (Comparative Fit Index), and SRMR (Standardized Root Mean Square Residual), considering commonly accepted thresholds for each [32,34]. Additionally, the reliability of the dimensions was examined using Cronbach’s alpha and McDonald’s omega coefficients, allowing for the determination of the internal consistency of each subscale of the instrument, ensuring that the items coherently measured the associated constructs [35,36]. Finally, academic results obtained by students in institutional transversal courses were integrated into the model to evaluate their performance concerning ERS competencies [33], (Fornell & Larcker, 1981). These results enable the establishment of empirical links between the appropriation of these competencies and academic performance in the aforementioned transversal training spaces, providing evidence on the formative coherence of the mesocurriculum.

To situate this research within a broader field, Table 1 presents a synthesis of relevant instruments and frameworks developed to measure ethics, social responsibility, and sustainability in higher education. While tools such as the Social Responsibility Questionnaire (SRQ), the Sustainability Literacy Test (Sulitest), and the Sustainability Awareness Questionnaire have contributed to assessing specific dimensions, they tend to focus on isolated aspects and lack an integrative perspective that articulates these three domains within a comprehensive competency framework.

**Table 1:** Synthesis of ERS frameworks in higher education and identified gaps.

Ref.	Instrument/ Framework	Description/ Purpose	Key Findings/ Evidence	Gap addressed by the present study
[37]	Sulitest (Sustainability Literacy Test)	Open, online test/training tool (UN collaboration) to assess sustainability literacy in HE.	Pilot suggests Sulitest not only measures literacy but can stimulate reflection, interest, and motivation in sustainability.	Focuses on sustainability literacy only; does not integrate ethics & social responsibility nor model predictive relations. Our study integrates ERS as a higher-order construct and links it to academic performance via SEM.
[38]	SCQ (Sustainability Consciousness Questionnaire)	Measures sustainability consciousness across Knowing–Caring–Acting dimensions.	CFA-supported structure; cross‑national adaptations show acceptable reliability/validity.	Strong on sustainability attitudes/behaviors but lacks explicit ERS higher‑order integration and outcome modeling. We validate ERS as a higher‑order construct and test predictive links with performance.
[39]	SRQ (Social Responsibility Questionnaire)	Assesses social responsibility beliefs/intentions in university students.	EFA/CFA evidence with acceptable reliability.	Covers SR only; no ethics/sustainability integration or SEM with outcomes. We embed SR as one dimension within ERS and estimate hierarchical SEM with academic outcomes.
[40]	Sustainability Perception Scale (36 items)	Quantitative questionnaire to measure sustainability perceptions among university students in Spain.	Sample of 285 students; exploratory and confirmatory factor analyses supported the 36-item scale; evidence of reliability and validity.	Captures general perceptions of sustainability only; does not integrate ethics and social responsibility nor estimate predictive academic outcomes. Our study builds a hierarchical ERS construct validated through SEM and linked to student performance.
[41]	Instrument for Sustainable Development in Education 5.0	Developed to assess academicians’ and students’ perceptions of sustainability practices in Higher Education Institutions (HEIs), aligned with Education 5.0 principles. It evaluates governance, human rights and gender perspective, environment, workplace experiences, and economic/social impact.	Content validity was confirmed using Aiken’s V (>0.89), and internal consistency was high (Cronbach’s alpha = 0.937). An Exploratory Factor Analysis (EFA) refined the instrument to 18 items grouped into four factors.	While this instrument validates sustainability practices in HEIs broadly, it does not specifically address the integration of ethics, social responsibility, and sustainability (ERS) into curricula or their predictive relationship with student academic performance. The present study extends this gap by modeling ERS competencies as a hierarchical construct and linking them to learning outcomes in transversal courses.
[42]	DIT‑2 (Moral Reasoning)	Assesses moral reasoning using scenario-based dilemmas (neo‑Kohlbergian).	Widely used; strong construct validity for ethical judgment.	Ethics‑focused; not tailored to ERS nor linked to sustainability/SR or curricular outcomes. We embed ethics within a comprehensive ERS model connected to curriculum and performance.
[15]	ERS Self‑perception Instrument	Develops and validates a three‑factor ERS instrument (Ethics, SR, Sustainability) with expert content validity (Aiken’s V) and pilot testing.	EFA/CFA; good reliability; supports three‑factor structure.	Does not estimate hierarchical SEM nor predictive links with academic performance. The present study extends Phase‑1 by testing a hierarchical SEM and linking ERS to transversal course performance.

The previous study [15] provided a first step by validating an instrument with strong psychometric properties; however, it did not incorporate structural relationships with academic performance. The present study advances this line of research by proposing and validating a hierarchical structural equation model (SEM) that not only ensures the reliability and validity of the measurement structure but also examines the predictive influence of ERS competencies on student performance in transversal courses. SEM is particularly appropriate because it overcomes the limitations of previous approaches, which relied mainly on exploratory or confirmatory factor analyses without modeling the interrelationships between higher- and lower-order constructs or their impact on educational outcomes. In this way, this research provides stronger empirical evidence to understand how ERS competencies function as integrated constructs in higher education.

Furthermore, this study explicitly aligns with Sustainable Development Goal (SDG) 4.7, which calls for ensuring that learners acquire the knowledge and skills needed to promote sustainable development, including education for sustainable lifestyles, human rights, gender equality, the promotion of a culture of peace, and global citizenship. By providing a validated model that links ethics, social responsibility, and sustainability with academic performance, this study offers a practical contribution to the assessment of institutional efforts to achieve SDG 4.7 in university contexts.

Based on the literature summarized in Table 1, previous instruments tend to assess isolated aspects of ERS and rarely integrate ethics, social responsibility, and sustainability into a single higher-order competency model. Moreover, most studies establish measurement validity (EFA/CFA) but do not examine structural relationships with educational outcomes or integrate assessments into mesocurricular spaces.

## Methods

To address the objectives of this study, a methodology was designed within the quantitative paradigm, adopting a non-experimental approach and oriented toward the psychometric analysis of a self-perception instrument focused on competencies in ethics, social responsibility, and sustainability (ERS) in the context of higher education. The methodological strategy initially involved the validation of the instrument through exploratory and confirmatory factor analyses. Subsequently, academic performance data from institutional transversal courses were integrated into the model in order to empirically examine the relationship between student performance and the appropriation of ERS competencies. This approach made it possible to evaluate the structural coherence of the model and its capacity to explain the degree of integration of these competencies in shared learning spaces.

### Instrument

The instrument used in this study is a questionnaire consisting of 30 items, structured into three distinct factors: Ethics, Social Responsibility, and Sustainability. This questionnaire was developed and refined through a content validation process, employing expert judgment, a recognized technique for validating measurement instruments. This process ensures that each item of the questionnaire is pertinent and suitable for assessing the specific competencies it aims to measure. Experts from various fields related to these topics evaluated each item in terms of clarity, relevance, and representativeness with respect to each factor, thus ensuring that the questionnaire reliably and validly reflects the proposed theoretical dimensions [15].

This study is an extension of the aforementioned work, which represented the first phase of a broader research project. In that initial phase, the instrument was validated by 21 expert judges with professional backgrounds in both higher education and the productive sector, contributing perspectives from academia and applied practice. Their professional experience ranged from 5 to 20 years, and their academic qualifications included 13 with a Master’s degree and 8 with a PhD, most with more than ten years of combined academic and professional experience. Content validity was assessed through two evaluation rounds using Aiken’s V index, which confirmed the clarity, relevance, and pertinence of the items. The results showed that all items exceeded the recommended threshold (V ≥ 0.70), with values ranging from 0.78 to 0.91, supporting their inclusion in the final version of the instrument [15].

Based on the experts’ feedback, redundant items were eliminated and the wording of several items was refined. The present manuscript builds on this pilot validation by extending the analysis through structural equation modeling to evaluate the relationships among ethics, social responsibility, and sustainability competencies in higher education.

This rigorous methodology contributes to the precision of the instrument, allowing the responses obtained from students to provide valid and actionable insights for educational research and pedagogical practices related to ethics, social responsibility, and sustainability in higher education. These three concepts are interconnected pillars that form the foundation for responsible practices and decisions [23].

In engineering, ethics is approached as a critical reflection on the moral decisions that professionals and organizations face in this field. It involves not only compliance with norms and regulations but also delves deeper into acting with integrity and moral responsibility. The ethical approach in engineering is crucial to ensuring that the actions and decisions of engineers consider their impacts on society and the environment [15,43].

Social Responsibility involves a proactive vision that seeks to con-tribute to the common good in a sustainable manner, ensuring that engineering activities not only seek technical or economic benefits but also promote social justice and respect for diversity. It is essential that engineering programs integrate social responsibility as a fundamental component of academic training, preparing students to effectively face and solve social and environmental challenges [15,44].

Sustainability is defined as a dynamic and balanced process that seeks to improve the quality of life through the conscious management of natural and human resources. In engineering, this means designing and operating in a way that minimizes negative impacts on the environment and society, promoting efficient resource use, energy conservation, and sustainable innovation [45].

### Population and sample

The study population consists of engineering students enrolled at a public university in Antioquia, Colombia. The sample includes 418 students. The descriptive analysis of the dataset reveals the composition of the students by gender, age, socioeconomic status, and semester. In terms of gender, the majority are male (361), followed by female (52) and other (5). The age distribution shows that most students are in the 15–25 age range (295), followed by those between 26 and 35 years (97), 36–45 years (24), and over 46 years (2). Regarding socioeconomic status, stratum 2 predominates with 193 students, followed by stratum 3 with 123, stratum 1 with 96, and stratum 4 with only 6. As for the academic semester, most of the students are in the first semester (215), with a significant decrease in subsequent semesters, notably the sixth semester with 84 students and the fifth with 37. These data provide a comprehensive view of the demographic and academic structure of the student sample in question. We defined the sample size using the criteria recommended by [46], and approximately 17 cases per item were considered.

### Ethical considerations

This research complied with the ethical principles established for studies involving human participants. The study protocol was reviewed and approved by the Research Ethics Committee of the Instituto Tecnológico Metropolitano (ITM), Act No. 02, April 2025. All participants were adults and voluntarily recruited through a digital link. Before accessing the questionnaire, participants read a digital informed consent form, which described the study objectives, procedures, voluntary participation, the academic purpose of the data, and measures to guarantee confidentiality. Only those who checked the option “I give consent” were able to proceed.

To safeguard confidentiality and anonymity, no personal identifiers were collected at any point. Data were coded and stored in institutional repositories with restricted access to the research team only. Academic performance indicators were obtained directly from institutional databases and linked to survey responses by numerical codes in the same order of reception, without access to student names or IDs. Data collection took place between May 20 and June 5, 2024. The researchers obtained the participants’ final grades in transversal courses related to ethics, social responsibility, and sustainability, in the same order in which the questionnaire responses were received. At no point did the researchers access any information that could directly identify the participants. Data access occurred on April 9, 2025, exclusively for research purposes and under institutional safeguards. These procedures ensured that all information was treated according to ethical standards of data protection and privacy.

### Data analysis

We provided the necessary instructions to ensure that all participants were fully informed about the study they were about to partake in. Each involved student received access to the informed consent document, which extensively outlines the study’s purpose, the procedures to follow, the expected benefits, and the potential risks. They had the opportunity to ask questions and were requested to accept and sign as a confirmation of their understanding and voluntary agreement to participate. This process is crucial to ensure that participation in the research is based on an informed decision, thereby respecting the fundamental ethical principles of autonomy and respect for individuals.

Data analysis for this study was conducted using the R statistical software, an open-source programming environment widely used for statistical analysis and graphics [47]. Specifically, the libraries lavaan, lavaanPlot, and psych were utilized for this analysis. The lavaan library is a tool for performing structural equation modeling [48], while lavaanPlot [49] complements lavaan by providing functionalities to visualize structural equation models, facilitating the interpretation and presentation of the results. On the other hand, the psych library is widely used in psychometrics and behavioral sciences, offering procedures for exploratory and confirmatory factor analysis, as well as other useful functions for psychometric analysis [50].

We conducted an Exploratory Factor Analysis (EFA) to investigate how items are interrelated, grouped into factors, and aligned with predefined theoretical hypotheses. Various statistical tests were employed for this analysis. Among these, the Kaiser-Meyer-Olkin (KMO) test for sampling adequacy is crucial as it assesses the suitability of data for factor analysis by evaluating the magnitude of partial correlations among items, excluding the influence of other items. KMO values range from 0 to 1, with values closer to 1 indicating better adequacy. We utilized the principal components method for the EFA, applying a varimax rotation to enhance the interpretability of the factors [51]. Varimax rotation provides a clearer distinction between factors. From a statistical perspective, the significance of factor loadings was evaluated; items with loadings above 0.45 were considered significant and acceptable for our analysis purposes [52].

Factor loadings represent the correlation between observed variables and latent factors; high values (generally greater than 0.4 or 0.5) indicate a strong association between a variable and a particular factor, facilitating the interpretation of the factors [53]. Communalities show the proportion of variance for each variable that is explained by all the factors extracted in the model. Higher communalities suggest that the factorial model captures the characteristics of the variables well [54]. Finally, the percentage of explained variance provides a measure of how much of the total variability in the dataset is attributed to each factor. Generally, the extracted factors should explain a large part of the total variance (often a combined total explained variance of 60% or more is considered adequate) [34,52].

After establishing the correlation between the constructs represented by the factors and having measured the observable variables through the scale items, we proceeded to estimate the model parameters. This calculation was carried out using a Confirmatory Factor Analysis (CFA), employing the Maximum Likelihood (ML) method. To ensure the accuracy of our results, we used bootstrap techniques that facilitate the estimation of the standard errors of the model parameters, regardless of the data distribution. This approach allows us to better fit the model to the actual structure of the observed data. In the goodness-of-fit analysis, we differentiated between global fit and incremental fit. Within the global fit, indicators such as the Root Mean Square Error of Approximation (RMSEA) and the Standardized Root Mean Square Residual (SRMR) were considered, which are essential for assessing the adequacy of the model. An RMSEA between 0.00 and 0.05 indicates a good fit [32], and values below 0.08 are considered acceptable [55], providing a robust framework for interpreting the effectiveness of the model in capturing the proposed structural relationships.

Incremental fit of a model is measured using specific indices such as the Tucker-Lewis Index (TLI), also known as the Non-Normed Fit Index (NNFI), and the Comparative Fit Index (CFI) [56]. These indicators evaluate the improvement of the proposed model compared to a null model, which assumes that there is no relationship between the variables. For the incremental fit to be considered satisfactory, these indices should approach or exceed the threshold of 0.90. In addition to these indices, the goodness of fit of the model is also verified by the chi-square (X^2^) ratio divided by the degrees of freedom, where a value between 0 and 2 generally indicates a good fit of the model (Hair et al., 2013). This range suggests that the model adequately fits the data without overfitting, which is crucial for validating the applicability and robustness of the model in capturing the dynamics of the studied variables.

Confirmatory Factor Analysis (CFA) provides key insights for assessing convergent validity, which verifies whether items are effectively associated with their respective constructs. This assessment involves analyzing the statistical significance of the factor loadings of the items in each latent construct [34,52]. Regarding the reliability of the instrument, Cronbach’s alpha was used to evaluate the internal consistency of the responses. A Cronbach’s alpha value of 0.7 or higher is generally accepted as indicative of good reliability [35,36,57]. McDonald’s reliability coefficient was calculated to contrast with Cronbach’s alpha [58].

Finally, the academic results obtained by students in two institutional transversal courses—one focused on environmental management and the other on the relationship between science, technology, and society—were incorporated into the model as indicators of performance in the competencies of ethics, social responsibility, and sustainability (ERS). This integration enabled a differentiated analysis of how each learning space contributes to the development of these competencies. By evaluating the model’s goodness-of-fit [56] indices in each case, the robustness of the proposed model was confirmed, providing empirical evidence of the coherence between academic performance and the appropriation of ERS competencies. These findings support the mesocurricular approach by showing that transversal courses play a strategic role in promoting ethical, socially responsible, and sustainable education.

## Results

This section presents the results of the exploratory and confirmatory factor analyses and the integration of students’ academic performance from two institutional transversal courses into the proposed theoretical model.

### Exploratory Factor Analysis (EFA)

The exploratory factor analysis revealed a structure composed of three latent factors. Most items associated with MR1 and MR2 showed standardized factor loadings above 0.60, indicating strong saturation and alignment with their respective dimensions. By established methodological criteria, items from these two factors with loadings below 0.60 were excluded from the model, as such values are generally interpreted as weak indicators of latent constructs [52]. In contrast, factor MR3 retained its four associated items despite having moderate loadings ranging from 0.49 to 0.59. This decision is based on the theoretical recommendation that a factor may be considered stable and interpretable if it includes at least three or four items with consistent, albeit moderate, loadings [59]. This process ensured the structural validity of the model while preserving its conceptual coherence.

**Fig 1:**
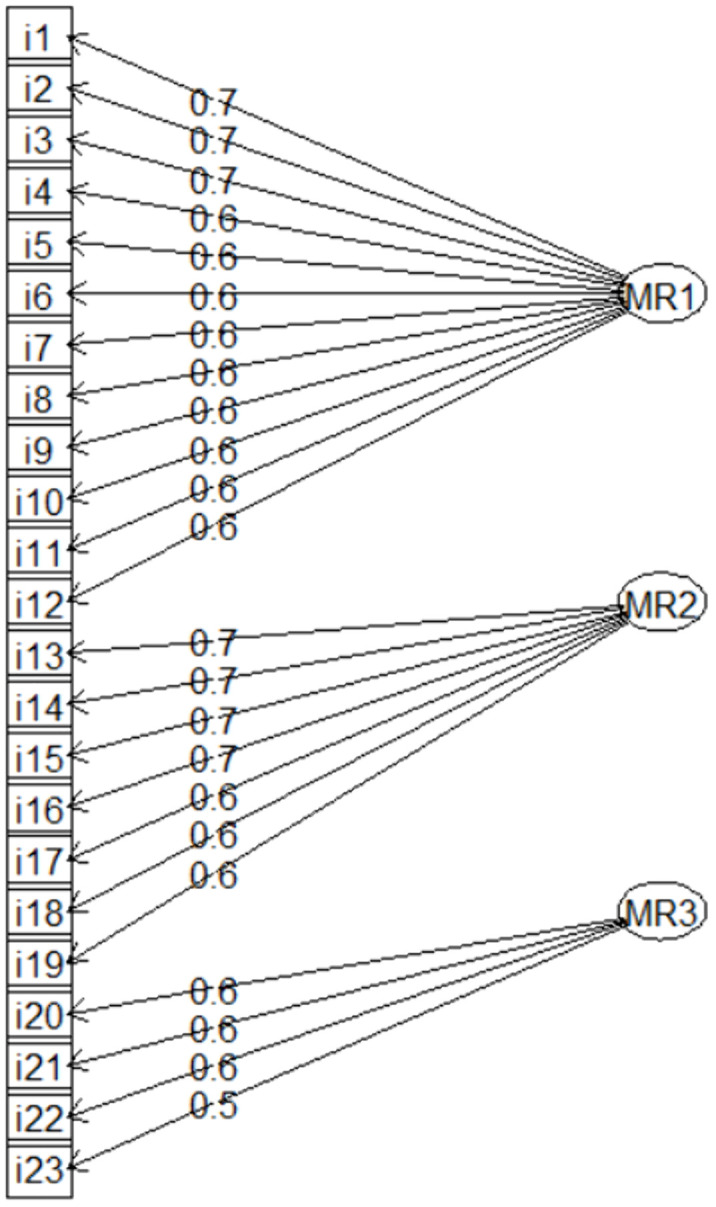
Factor loadings obtained through exploratory factor analysis after item refinement.

After eliminating the items with factor loadings below the established threshold and reordering them, a new exploratory factor analysis (EFA) was performed. Fig 1 displays the resulting factor loadings for each item, grouped according to the factors defined in the conceptual model: MR1 (Ethics), MR2 (Sustainability), and MR3 (Social Responsibility).

Sampling adequacy was excellent, with an overall KMO index of 0.94 and individual item values above 0.90, indicating strong partial correlations among variables and supporting the suitability of factor analysis. Bartlett’s test of sphericity was significant (p < 0.001), confirming that the correlation matrix significantly differs from the identity matrix [52]. The three factors jointly explained 46% of the total variance, with MR1 accounting for 22%, MR2 for 16%, and MR3 for 9%. Factor loadings ranged from 0.56 to 0.72 for most items, suggesting a clear and well-defined structure. Factor score adequacy was high, particularly for MR1(Ethics) and MR2 (Sustainability), with correlations of 0.93 and 0.89, respectively, between the estimated scores and the underlying latent factors.

### Confirmatory Factor Analysis (CFA)

**Fig 2:**
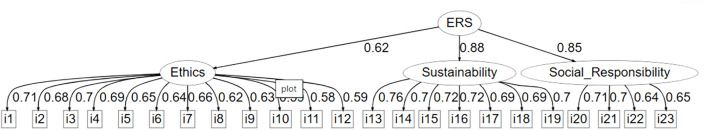
Flow diagram of the proposed model.

To verify the relationships among the dimensions and to confirm whether the items align with the proposed model, a confirmatory factor analysis (CFA) was conducted using the maximum likelihood (ML) estimation method and applying the model development strategy. Fig 2 provides a graphical representation of the proposed structural equation model (SEM), illustrating the relationships among latent and observed variables, as well as the structural paths specified in the confirmatory analysis.

The SEM model is hierarchical, in which the second-order latent variable ERS (Ethics, Social Responsibility, and Sustainability) explains three first-order latent dimensions: Ethics, Sustainability, and Social Responsibility. The relationships between ERS and the three dimensions are strong, with standardized loadings of 0.88 for Sustainability, 0.85 for Social Responsibility, and 0.62 for Ethics. This indicates that the ERS competency is more strongly manifested through the components of sustainability and social responsibility. Each latent variable is measured by several observed items (i1–i23), with standardized factor loadings ranging from 0.58 to 0.76, suggesting adequate internal consistency. Specifically, the variable Ethics is represented by items i1 to i12, with loadings between 0.58 and 0.71; Sustainability (i13–i19) shows loadings between 0.69 and 0.76; and Social Responsibility (i20–i23) exhibits loadings ranging from 0.64 to 0.71. These loadings indicate that the items are well aligned with the dimensions they are intended to measure, especially in the case of Sustainability and Social Responsibility, where the loadings are generally higher and more homogeneous [52]. The model presents a structure that is theoretically coherent, in which the three dimensions contribute significantly to the ERS competency as a second-order construct.

The goodness-of-fit indices obtained are presented in Table 2 and, as mentioned, include the Tucker-Lewis Index (TLI), also known as the Non-Normed Fit Index, and the Comparative Fit Index (CFI). Both indices range from 0 to 1, where 1 indicates a perfect fit and values below.90 suggest the need to revise the model. Additionally, the Root Mean Square Error of Approximation (RMSEA) and the Standardized Root Mean Square Residual (SRMR) were included. Other fit indices were calculated under the assumption that the specified model is correct, including the LO 90 and HI 90 confidence intervals. The SRMR is considered to indicate good fit when ranging from 0.00 to 0.05, and acceptable fit when it falls between 0.05 and 0.08 [32,55]. The results of these fit indices are presented in Table 2 and reflect the underlying data structure.

**Table 2:** Model’s goodness-of-fit indices.

X^2^	df	X^2^/ df	TLI	CFI	SRMR	RMSEA	LO 90	HI 90
438.021	227	1.929	0.940	0.946	0.042	0.047	0.040	0.054

**Fig 3:**
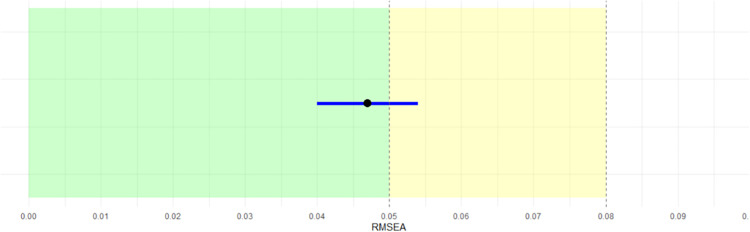
RMSEA value with 90% confidence interval.

The goodness-of-fit indices obtained from the analysis indicate that the model fits the observed data well. The chi-square statistic divided by its degrees of freedom (χ²/df) yielded a value close to 2, reflecting a reasonable approximation error. Moreover, both the Tucker-Lewis Index (TLI) and the Comparative Fit Index (CFI) exceeded the recommended threshold of 0.90, suggesting an excellent fit compared to the null model. Additionally, the 90% confidence interval for the RMSEA, ranging from 0.040 to 0.054, further supports a very good to acceptable model fit, with a high likelihood that the population RMSEA does not exceed the conventional cutoff of 0.08. Fig 3 represents the point estimate of the RMSEA along with its confidence interval.

The lower bound of the interval falls well below 0.05, and the upper bound remains within the acceptable range, reinforcing the conclusion that the model achieves an adequate approximation of the population covariance structure [60].

Convergent validity was assessed by examining the statistical significance of the standardized factor loadings for each item associated with its respective latent factor. A standardized factor loading of 0.60 or higher, along with a Critical Ratio (CR) greater than 1.96 (p < .05), is considered evidence of adequate convergence. As shown in Table 3, all items meet these criteria, with both their standardized loadings and corresponding CR values falling within the acceptable range.

**Table 3:** Standardized factor loadings and critical ratios.

Item	Standardized factor loading	CR	Significance
i1	0.706		
i2	0.681	13.051	<.001
i3	0.697	13.347	<.001
i4	0.692	13.247	<.001
i5	0.652	12.497	<.001
i6	0.642	12.315	<.001
i7	0.662	12.686	<.001
i8	0.621	11.924	<.001
i9	0.627	12.033	<.001
i10	0.592	11.375	<.001
i11	0.584	11.222	<.001
i12	0.589	11.326	<.001
i13	0.76		
i14	0.697	14.162	<.001
i15	0.723	14.742	<.001
i16	0.719	14.632	<.001
i17	0.692	14.041	<.001
i18	0.687	13.94	<.001
i19	0.7	14.214	<.001
i20	0.705		
i21	0.695	12.011	<.001
i22	0.638	11.176	<.001
i23	0.654	11.425	<.001

The items used as reference indicators (such as i1, i13, and i20) do not have CR values or significance levels because their parameters were fixed for model identification purposes. Table 4 presents the items from the self-perception questionnaire related to ERS (Ethics, Social Responsibility, and Sustainability).

**Table 4:** Items obtained from the exploratory factor analysis and their corresponding dimensions.

No	Items	Dimensions
i1	I am willing to accept the consequences of my mistakes in my daily actions.	Ethics
i2	Doing what is right in my daily behavior allows me to be at peace with myself.	
i3	Working with passion is part of my personal fulfillment.	
i4	I convey my own values through my daily actions.	
i5	I consider it worthwhile to accept the risk of making mistakes if it helps improve my performance in my field of study.	
I6	To avoid making mistakes in my career, I must be aware of the limits of my knowledge and skills.	
i7	I consider it essential to take ethical aspects into account in my academic and future professional career.	
i8	Fulfilling my commitments on time is important in my daily conduct.	
i9	I am willing to spend time updating my knowledge on any aspect of my field.	
i10	I should not make important decisions without first considering their consequences.	
i11	For good performance in my career, I cannot limit myself to developing only technical skills.	
i12	Maintaining confidentiality is important in daily practice.	
i13	I recognize the potential of the human and natural resources in my environment for use in sustainable development.	Sustainability
i14	I am capable of imagining and anticipating the impacts of environmental changes on social and economic systems.	
i15	I am aware of the importance of sustainability in society, and I learn from and influence the community in which I live.	
i16	I use resources sustainably to prevent negative impacts on the environment and social and economic systems.	
i17	I create and contribute solutions from a critical and creative perspective on issues of technology and engineering, considering sustainability.	
i18	I analyze situations individually or in groups regarding sustainability and its relationship with society, the environment, and the economy, both locally and globally.	
i19	I am aware of and concerned about local problems and their relationship with national and global factors.	
i20	As a student, I feel I have the tools to contribute to social, political, and economic changes in my environment.	Social Responsibility
i21	As a student, I would like to influence public policies that improve the quality of life for minority groups (race, ethnicity, sexual orientation) and vulnerable groups (children, women, elderly people).	
i22	I believe that my education provides me with tools to monitor public or private programs and initiatives aimed at social transformation.	
i23	I believe that through my professional practice I can contribute to reducing poverty and inequality in my region.	

### Reliability

The reliability of the scales was assessed for both the higher-order and lower-order constructs using Cronbach’s alpha and McDonald’s omega. For the higher-order construct (ERS), Cronbach’s alpha was α = 0.914 and McDonald’s omega was ω = 0.920, both indicating excellent internal consistency and supporting the coherence of the items in measuring the same underlying concept. For the lower-order constructs, Ethics showed α = 0.893, ω = 0.905, Sustainability α = 0.876, ω = 0.892, and Social Responsibility α = 0.766, ω = 0.745. All these values demonstrate satisfactory to excellent reliability, consistent with commonly accepted thresholds, where coefficients above 0.70 are generally considered indicative of adequate internal consistency. The inclusion of McDonald’s omega provides a more flexible estimate of reliability, as it does not assume equal item loadings, thereby complementing Cronbach’s alpha in assessing scale consistency [35,36,57,61].

### Integration of academic performance into the structural model

**Fig 4:**
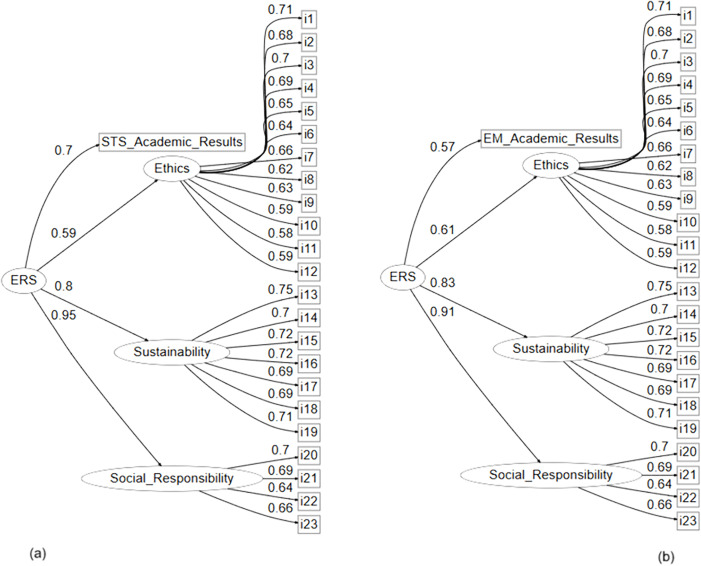
SEM model integrating the (a) Environmental Management dimension and (b) Socio-Scientific dimension.

Fig 4 presents the previously described model, now incorporating academic performance data from institutional transversal courses.

This extension allows for an empirical analysis of the relationship between ethics, social responsibility, and sustainability (ERS) competencies and students’ academic performance in learning contexts focused on environmental management (a) and the socio-scientific dimension (b).

Model (a) incorporates the academic performance results from a course related to science, technology, and society (STS). The ERS competency is shown to have a positive influence on the constructs of Ethics, Sustainability, and Social Responsibility, with standardized coefficients of 0.59, 0.80, and 0.95, respectively. Additionally, ERS significantly predicts academic performance in the transversal course focused on socio-scientific education, with a standardized coefficient of 0.968 (p < .001), Highlighting its relevance as a key competency in university education.

Model (b) of the structural equation modeling (SEM) framework examined the relationship between ethics, social responsibility, and sustainability (ERS) competencies and students’ academic performance in a course related to environmental management. The higher-order construct ERS significantly predicted the first-order factors (Ethics, Sustainability, and Social Responsibility) and was also a significant predictor of academic performance in the aforementioned course, with a standardized coefficient of 0.574 (p < .001). These findings support the theoretical validity of the ERS construct and its relevance in institutional courses focused on sustainability.

Table 5 summarizes the main goodness-of-fit indices for models (a) and (b), which incorporate academic performance data from transversal courses related to Science, Technology, and Society (STS) and Environmental Management, respectively. These indicators allow for the evaluation of each model’s fit to the data.

**Table 5:** Goodness-of-fit indices of models (a) and (b).

Model	X^2^	df	X^2^/ df	TLI	CFI	SRMR	RMSEA
(a)	483.763	249.000	1.942	0.937	0.943	0.046	0.047
(b)	474.792	249.000	1.906	0.938	0.944	0.043	0.047

Models (a) and (b) demonstrated adequate goodness-of-fit indices, supporting the structural validity of the proposed relationships. In both cases, the CFI (Comparative Fit Index) and TLI (Tucker-Lewis Index) exceeded the recommended threshold of 0.90, with values of 0.944 and 0.938 for Model (b), and 0.943 and 0.937 for Model (a), indicating satisfactory comparative fit relative to the null model.

The RMSEA (Root Mean Square Error of Approximation) was 0.047 in both models. Likewise, SRMR (Standardized Root Mean Square Residual) values were low in both cases (0.043 and 0.046), falling within the range considered indicative of good fit (< 0.08). Overall, these results reflect a well-specified theoretical structure that is consistent with the observed data in both educational contexts.

## Discussion

The results of the exploratory factor analysis reveal a well-defined three-factor structure that explains 46% of the total variance, with most standardized factor loadings exceeding 0.60, indicating adequate item saturation. The overall KMO index (0.94) and the significance of Bartlett’s test of sphericity (χ² = 5588.429, p < .001) confirm the suitability of applying factor analysis to the data. These findings are consistent with methodological guidelines suggesting that factors should be retained when cumulative explained variance exceeds 40% and loadings are greater than 0.60 to ensure the instrument’s convergent validity [62]. In particular, the sample adequacy and clarity of the loadings support the conclusion that the items are theoretically well aligned with the underlying factors, reinforcing the empirical foundation of the proposed measurement model.

The structural equation model (SEM) showed a good fit to the data, with indices supporting the validity of the proposed model. The chi-square statistic was significant (χ² = 438.021, df = 227, p < .001), which is expected in large samples; however, this was offset by complementary goodness-of-fit indicators: CFI = 0.946 and TLI = 0.940, both exceeding the recommended threshold of 0.90 [32]. The RMSEA was 0.047, and the SRMR was 0.042, suggesting that the model approximates the observed data well. In the measurement model, all standardized factor loadings were statistically significant (p < .001) and above 0.58, indicating adequate convergent validity. The higher-order construct ERS significantly explained the three first-order factors: Ethics (0.620), Sustainability (0.884), and Social Responsibility (0.847), confirming its structural consistency. Overall, the results provide empirical support for the hierarchical structure of the model and highlight its relevance for assessing ERS competencies in university contexts [32,52].

The reliability analysis provided robust evidence for the internal consistency of both the higher-order and lower-order constructs. The ERS higher-order construct yielded a Cronbach’s alpha of 0.914 and McDonald’s omega of 0.920, reflecting excellent consistency among the items and supporting the theoretical coherence of the multidimensional competency. Likewise, the lower-order dimensions also showed satisfactory reliability: Ethics (α = 0.893; ω = 0.905), Sustainability (α = 0.876; ω = 0.892), and Social Responsibility (α = 0.766; ω = 0.745). All values exceeded the commonly accepted threshold of 0.70 in social sciences, confirming the adequacy of the item groupings under each factor. The joint reporting of alpha and omega further reinforces the robustness of the scales, since omega does not assume equal factor loadings and thus complements Cronbach’s alpha in evaluating internal consistency [36,57,61]. These results support the use of the instrument in higher education contexts where measuring transversal competencies is essential for curriculum development and evaluation.

Structural models (a) and (b) demonstrated a good fit to the data, supporting the hierarchical validity of the ERS competency across different curricular contexts. In both models, the fit indices were satisfactory, with CFI values above 0.94, TLI near 0.94, RMSEA = 0.047, and SRMR values below 0.05, indicating a solid approximation of the model to the observed data. The second-order ERS construct significantly explained the three first-order dimensions in both models, with standardized loadings ranging from 0.59 to 0.95, and particularly strong associations with Sustainability and Social Responsibility. Additionally, ERS showed a significant predictive relationship with academic performance in both transversal courses: STS (model (a)) with a standardized coefficient of 0.698, and Environmental Management (model (b)) with 0.574 (p < .001 in both cases) [32]. These findings suggest that the development of ethics, sustainability, and social responsibility competencies is positively associated with academic achievement in mesocurricular learning contexts that emphasize socio-scientific reasoning and sustainability, aligning with educational frameworks that promote key competencies for sustainable development.

The results of the hierarchical model not only confirm the structural validity of the ERS competency but also provide empirical evidence of its effective integration within transversal learning spaces framed by a mesocurricular context. The significant influence of the second-order ERS construct on the dimensions of Ethics, Sustainability, and Social Responsibility supports its conception as an integrative competency, essential for higher education oriented toward social transformation. The inclusion of transversal courses as mesocurricular components enables the operationalization of these competencies within the curriculum, promoting their development from a situated perspective that aligns with institutional policies. These results are consistent with university education proposals that advocate for strengthening competencies for responsible citizenship and sustainability in higher education settings [63,64]. Overall, the model offers not only psychometric robustness but also a theoretical foundation applicable to the assessment and integration of ERS competencies in university curriculum design.

### Limitations

Despite the robustness of the findings, several limitations should be acknowledged. First, the study relied on a single-institution sample of students, which may restrict the generalizability of the results to other disciplines and higher education contexts. Second, data were collected through self-reported questionnaires, which may be subject to response biases such as social desirability. Finally, although the model demonstrated strong psychometric properties, its applicability in different cultural and institutional settings remains to be further tested. These limitations highlight the need for caution when extrapolating the results, and suggest directions for future research aimed at strengthening the external validity of the ERS competency model.

### Unexpected findings

A noteworthy finding of this study was that the Ethics dimension exhibited a weaker structural influence compared to Sustainability and Social Responsibility. This result was not fully aligned with our initial expectations, since ethics is often considered a foundational component of ERS frameworks. One possible explanation is that, within the analyzed curriculum, sustainability and social responsibility are more explicitly integrated into transversal courses, while ethics tends to appear in a more fragmented or implicit manner.

The lower structural weight of Ethics (0.620) compared to Sustainability (0.884) and Social Responsibility (0.847) warrants a deeper interpretation. One possible explanation is that institutional curricular strategies have prioritized sustainability and social responsibility, especially through transversal courses focused on environmental management and socio-scientific reasoning, which are strongly aligned with SDG 4.7. In contrast, ethics may be less visible or formally articulated within these mesocurricular spaces, often embedded in broader discourses without a clear evaluation framework. Another possible factor is that students tend to perceive sustainability and social responsibility as more directly connected to practical and societal challenges, whereas ethics is often seen as more abstract. This finding points to the need for a more explicit integration of ethical dimensions in higher education curricula.

### Comparisons with previous studies

The psychometric properties of the proposed model are consistent with, and in some cases stronger than, those reported in previous literature. Our model achieved KMO = 0.94, CFI = 0.944, TLI = 0.938, SRMR = 0.043, and RMSEA = 0.047, all of which indicate a robust factorial structure and model fit. These results are comparable to those reported by other studies. For example, [40] found KMO = 0.898, RMSEA = 0.043, CFI = 0.972, TLI = 0.979, SRMR = 0.076, and Cronbach’s alpha above recommended thresholds. Similarly, [39] reported CFI = 0.95 and RMSEA = 0.06, while [38] obtained RMSEA = 0.041, CFI = 0.953, and TLI = 0.944, also demonstrating strong psychometric performance. In terms of reliability, the internal consistency indices of our instrument were robust: Cronbach’s alpha values for the subscales ranged from 0.766 to 0.893, while McDonald’s omega values ranged from 0.745 to 0.905. These results are consistent with or superior to those reported in related ERS instruments [41], thereby reinforcing both the reliability and the validity of the proposed model and highlighting its contribution to the field.

### Practical implications for higher education

The results of this study carry relevant practical implications. For curriculum design, the validated ERS model provides a framework for systematically integrating ethics, social responsibility, and sustainability into transversal and disciplinary courses. For assessment instrument development, the validated scale offers a reliable tool for universities to monitor ERS competencies in different contexts. Regarding lecturer training, the findings highlight the importance of strengthening faculty preparation in ethics education, ensuring that ethical reasoning is addressed as explicitly as sustainability and social responsibility. Finally, at the level of institutional policies, the hierarchical ERS model contributes evidence to support the alignment of higher education strategies with SDG 4.7, which emphasizes the promotion of knowledge, skills, and values for sustainable development and global citizenship.

## Conclusions

The results of this study provide strong empirical evidence for the validity and reliability of the proposed hierarchical model for assessing competencies in ethics, social responsibility, and sustainability (ERS) in university contexts. Confirmatory factor analysis and the goodness-of-fit indices support the theoretical structure of the model, while the high internal consistency values reflect an appropriate clustering of items within their respective dimensions. Moreover, the inclusion of transversal courses with a focus on sustainability and the socio-scientific dimension allowed for the verification of how these competencies are articulated within the mesocurricular framework, demonstrating their positive influence on students’ academic performance.

Taken together, these findings reinforce the need to explicitly integrate ERS competencies into higher education curricula, not merely as complementary elements, but as strategic components that promote the formation of critical, engaged citizens oriented toward sustainable development. In addition to its psychometric robustness, the model contributes theoretically by extending previous frameworks: it demonstrates that ERS can be conceived as a higher-order construct that not only integrates ethics, social responsibility, and sustainability, but also predicts academic achievement in transversal courses, thereby bridging the gap between competency assessment and learning outcomes.

Nevertheless, some limitations must be acknowledged. Methodologically, the study was restricted to engineering students from a single Colombian university, which limits the generalizability of the results. The reliance on self-reported measures may also introduce response bias. From a theoretical perspective, the model does not capture all possible dimensions of ERS, particularly contextual or cultural variations in ethical reasoning. Practically, the findings are bounded by the institutional context in which the study was conducted, and their applicability to other higher education systems should be tested.

Future research should address these limitations through more diverse and representative samples across different disciplines and international contexts to examine the cross-cultural validity of the model. Longitudinal studies are needed to evaluate the stability of ERS competencies throughout the student’s academic trajectory and their long-term impact on professional practice and social engagement. Comparative studies across disciplines could further determine whether the hierarchical structure of ERS competencies is consistent in non-engineering contexts. Another promising line of inquiry involves exploring the integration of active methodologies, such as project-based learning or service-learning, within mesocurricular frameworks, and evaluating their effectiveness in strengthening these key competencies for global and sustainable citizenship.

## Supporting information

S1 File DataR_EFA. (TXT)

S2 File DataR_Model. (TXT)

S3 File Scripts.R. (TXT)
